# Capture, Unfolding, and Detection of Individual tRNA Molecules Using a Nanopore Device

**DOI:** 10.3389/fbioe.2015.00091

**Published:** 2015-06-24

**Authors:** Andrew M. Smith, Robin Abu-Shumays, Mark Akeson, David L. Bernick

**Affiliations:** ^1^Department of Chemistry and Biochemistry, University of California Santa Cruz, Santa Cruz, CA, USA; ^2^Department of Biomolecular Engineering, University of California Santa Cruz, Santa Cruz, CA, USA; ^3^Genomics Institute, University of California Santa Cruz, Santa Cruz, CA, USA

**Keywords:** tRNA, nanopore, nanotechnology, RNA sequencing, single molecule

## Abstract

Transfer RNAs (tRNA) are the most common RNA molecules in cells and have critical roles as both translators of the genetic code and regulators of protein synthesis. As such, numerous methods have focused on studying tRNA abundance and regulation, with the most widely used methods being RNA-seq and microarrays. Though revolutionary to transcriptomics, these assays are limited by an inability to encode tRNA modifications in the requisite cDNA. These modifications are abundant in tRNA and critical to their function. Here, we describe proof-of-concept experiments where individual tRNA molecules are examined as linear strands using a biological nanopore. This method utilizes an enzymatically ligated synthetic DNA adapter to concentrate tRNA at the lipid bilayer of the nanopore device and efficiently denature individual tRNA molecules, as they are pulled through the α-hemolysin (α-HL) nanopore. Additionally, the DNA adapter provides a loading site for ϕ29 DNA polymerase (ϕ29 DNAP), which acts as a brake on the translocating tRNA. This increases the dwell time of adapted tRNA in the nanopore, allowing us to identify the region of the nanopore signal that is produced by the translocating tRNA itself. Using adapter-modified *Escherichia coli* tRNA^fMet^ and tRNA^Lys^, we show that the nanopore signal during controlled translocation is dependent on the identity of the tRNA. This confirms that adapter-modified tRNA can translocate end-to-end through nanopores and provide the foundation for future work in direct sequencing of individual transfer RNA with a nanopore-based device.

## Introduction

Transfer RNAs (tRNA) decode genetic information, delivering to the protein-synthesizing ribosome the individual amino acids specified by each codon of a messenger RNA. In light of this, it is unsurprising that they are the most numerous RNA species in the cell, composing approximately 80% of the RNA molecules per generation in yeast (Phizicky and Hopper, 2010). These small, non-coding RNA molecules contain numerous post-transcriptionally modified nucleotides, which contribute to tRNA fold stabilization, codon recognition, and aminoacylation [reviewed in Gustilo et al. (2008) and Motorin and Helm (2010)]. A growing body of research indicates that tRNAs and their nucleotide modifications are directly targeted for regulation as well as acting as potential regulators themselves [reviewed in Yi and Pan (2011) and Raina and Ibba (2014)].

Current methods for analysis of tRNAs, and RNA in general, include RNA-seq, microarray, and mass spectrometry. These methods are proven tools for detection of novel tRNAs and global tRNA expression patterns (Dittmar et al., 2006; Chan et al., 2011). However, each method has limitations.

High-throughput RNA sequencing (RNA-Seq) methods require extensive library preparation, including PCR amplification, to prepare cellular RNA for sequencing. A reverse transcription (RT) step is necessary to copy the original RNA sequence to cDNA, which results in loss of the original RNA strand. Additionally, the RT step is impeded by the occurrence of structure and nucleotide modifications, which are both commonly found in tRNAs. These “RT-stops” result in truncated cDNA. While these RT stops have been exploited by various methods to infer structure or nucleotide modification state in RNA, they do not permit direct detection of nucleotide sequence and modification identity along intact RNA (Omer et al., 2000; Merino et al., 2005; Bernick et al., 2012; Vilfan et al., 2013). Methods have been developed for detecting a few specific RNA modifications, such as bisulfite treatment for 5-methylcytosine or immunoprecipitation for N6-methyladenine, but these require additional labor-intensive steps (Schaefer et al., 2009; Dominissini et al., 2012).

Microarrays share a similar problem with loss of modification information and extended processing times. Success has been reported in detecting nucleotide modifications in tRNAs that affect hybridization of the target molecule to array probes, which limits detection to those modifications that affect Watson–Crick base-pairing (Hiley et al., 2005; Saikia et al., 2010). In addition, microarray probe design requires prior knowledge of the target sequence, which limits detection to known RNAs.

Liquid chromatography coupled to mass spectrometry (LC-MS) has also been applied to study tRNAs. In particular, two recent publications have used LC-MS to examine dynamic changes in tRNA modification state in *Saccharomyces cerevisiae* under varied environmental growth conditions (Chan et al., 2010, 2012). However, the nucleolytic fragmentation that is required by this method prevents observing modifications in the full sequence context of the tRNA.

Detection of tRNA subpopulations that result from variability in modification, as seen in mitochondrial diseases, will benefit from direct interrogation of intact, single tRNA molecules (Suzuki et al., 2011). In two specific mitochondrial examples, failed tRNA modifications cause dysfunctional translation of mitochondrial enzymes. These in turn result in the mitochondrial encephalopathies, myoclonic epilepsy with ragged-red fibers (MERRF), and mitochondrial encephalomyopathy, lactic acidosis, and stroke-like episodes (MELAS) (Yasukawa et al., 2001; Kirino et al., 2004). In these examples, a method that examines individual tRNA molecules could reveal the extent of incompletely modified or mutant forms in these complex disease phenotypes.

Nanopore sensors interrogate single molecules and should allow for examination of several thousand individual tRNA in a single experiment. Nearly 20 years ago, biological nanopores with 1–2 nm limiting apertures were conceived as single-molecule sensors for nucleic acids (Kasianowicz et al., 1996). In concept, the nucleotide sequence of an individual molecule would be read by observing changes in ionic current as the linearized strand is electrophoresed through the nanopore aperture. Recent developments in sensing DNA have coupled an enzyme to regulate DNA movement in single nucleotide steps through a nanopore, which produced ionic current traces that provide a single base readout of DNA sequence (Cherf et al., 2012; Manrao et al., 2012). Additionally, experiments by our group and others have shown that DNA cytosine modifications can be detected with high confidence from individual nanopore reads of chemically synthesized DNA (Laszlo et al., 2013; Schreiber et al., 2013). Applying these principles should provide a high-throughput method to directly examine tRNA nucleotide sequence.

With RNA, strand sequencing with single-nucleotide resolution has yet to be demonstrated with a nanopore device. However, Ayub and Bayley have shown that immobilized RNA strands within a modified α-HL nanopore apparatus yield distinct ionic current amplitudes that discriminate between canonical and select modified ribonucleotides (Ayub and Bayley, 2012). Further, solid-state nanopores with pore-sizes that exceed 3 nm have been shown to distinguish folded tRNA molecules from linear double-stranded RNA and DNA (Wanunu et al., 2010). By extension, these results suggest that nanopore sensors could detect sub-molecular features of tRNA, including nucleotide modifications, if tRNAs can be mechanically unfolded and electrically motivated to pass through the pore.

With this in mind, we sought to develop a mechanism to specifically capture tRNA molecules, promote their mechanical unfolding, and initiate threading of the linearized strand through the nanopore lumen. To this end, we designed an oligonucleotide adapter that can be attached to intact tRNAs. To slow tRNA translocation through the pore, we employed a non-catalytic protein “brake” that loads onto the adapter. This allowed us to determine the direction of strand translocation and provided sufficient resolution to determine ionic current signal features associated with the translocating adapter and the tRNA. Results presented here demonstrate that tRNA attached to such an adapter and modulated by a protein brake can be completely translocated through the α-HL nanopore, and that *Escherichia coli* tRNA^fMet^ and tRNA^Lys^ produce differentiable nanopore signals in this system. This provides the foundation for future work aimed at achieving single nucleotide resolution of individual transfer RNA.

## Materials and Methods

### Oligonucleotide synthesis and purification

All oligonucleotides were synthesized by the Stanford Protein and Nucleic Acids facility (PAN) using standard phosphoramidite chemistry. Oligonucleotides were purified by denaturing 7M urea polyacrylamide gel electrophoresis (PAGE) in 1× TBE, followed by overnight elution from an excised band at 4°C in 300 mM Sodium Acetate pH 5.2 and 1 mM EDTA. DNA was precipitated and recovered by adding 100% molecular biology grade ethanol (Sigma Aldrich) to 70% final v/v and centrifuged for 30 min at 14,000 × *g* and 4°C. Alternately, RNA-containing oligonucleotides were recovered by precipitation in 75% ethanol v/v and centrifuged at 14,000 × *g* for 30 min at 4°C. The ethanol mixture was aspirated and oligonucleotides were then washed with an equal volume of 70 or 75% ethanol and pelleted again for 10 min at 14,000 × *g* and 4°C. Ethanol was aspirated and the pellets were allowed to dry under vacuum to remove residual ethanol. Oligonucleotides were then resuspended in nuclease free water, quantified by Nanodrop (Thermo Scientific), and stored at −80°C.

### Adapter design and hybridization

**Figure 1:**
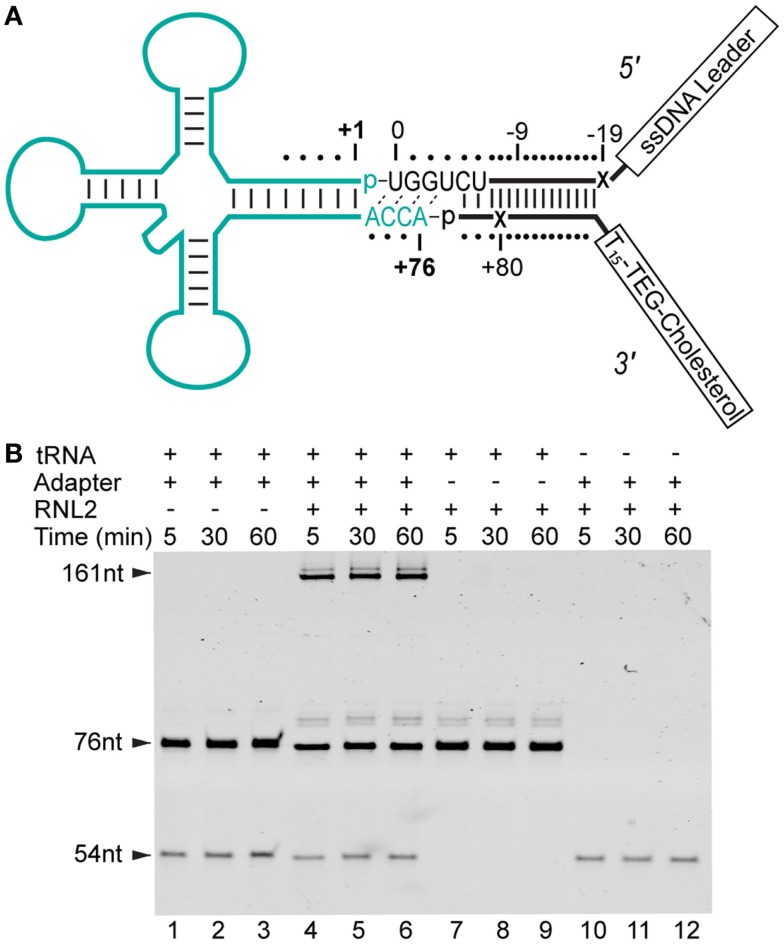
**Strategy for constructing adapter-linked tRNA molecules for nanopore experiments**. **(A)** The DNA/RNA chimeric adapter (black lines; RNA nucleotides black letters) was composed of a double stranded region and a four nucleotide RNA overhang (UGGU) ligated to the tRNA (cyan). The adapter specifically targets the conserved CCA tail of deacylated, native tRNA. Phosphodiester bonds formed by enzymatic ligation between the tRNA and the adapter are indicated by dashes between the 3′ terminal nucleotide and 5′ phosphate (p) at ligation junctions. The boxed tail regions were a single-stranded DNA (ssDNA) leader used for nanopore capture and a poly(dT) region with a terminal TEG-linked (triethylene glycol) cholesterol. This terminal TEG-cholesterol on the 3′ adapter strand was designed to localize substrate at the lipid bilayer. Tails are not drawn to scale. The adapter nucleotides are numbered relative to the first and last nucleotide of a canonical tRNA. The X’s indicate abasic positions. **(B)** Denaturing PAGE analysis of tRNA-adapter ligation reaction (see Materials and Methods). Time points are in minutes. Lanes 1–3, control reaction with *S. cerevisiae* tRNA^Phe^ (76 nt) and adapter (54 nt leader strand), but absent RNA Ligase 2 (RNL2). Lanes 4–6, *S. cerevisiae* tRNA^Phe^ incubated with the adapter and RNL2. Lanes 7–9, control reaction with RNL2 but absent adapter. Lanes 10–12, control reaction absent tRNA^Phe^. The 31 nt 3′ adapter oligonucleotide stains poorly (not shown). All subsequent nanopore experiments were conducted with complete ligation products after gel purification (see Materials and Methods).

The two oligonucleotides of the tRNA adapter were designed to form a partially double-stranded y-shape. The design included one end that targeted the ACCA tail of tRNA, while the other end contained unpaired single stranded regions (see Figure 1A). The adapter strands also included positions where abasic (1′-H deoxyribose) residues were incorporated to act as ionic current markers for the different ends of the adapted RNA molecule.

5′ leading strand oligonucleotide (bolded italicized bases indicate RNA; plain letters are DNA, X indicates an abasic 1′-H deoxyribose): 5′-CTCACCTATCCTTCCACTCATACTATCATTATCTXTCAGATCTCACTA***UCUGGU-***3′.

3′ trailing strand oligonucleotide (X indicates an abasic 1′-H deoxyribose; Z is a triethylene glycol-cholesterol): 5′-phos-GATXGTGAGATCTGATTTTTTTTTTTTTTTZ-3′.

For experiments to determine which end of the adapted RNA molecules entered the pore first, new adapter nucleotides were synthesized with one of the abasic markers (as indicated above) replaced by the nucleotide that restored complementarity in the double stranded portion of the adapter. In these experiments, one of the following oligonucleotides replaced its corresponding abasic-containing strand in the adapter:

5′ leading strand oligonucleotide: 5′-CTCACCTATCCTTCCACTCATACTATCATTATCTCTCAGATCTCACTA***UCUGGU***-3′.

3′ trailing strand oligonucleotide: 5′-phos-GATAGTGAGATCTGATTTTTTTTTTTTTTTZ-3′.

Adapters were formed by combining leading and trailing strands at 100 μM in 10 mM Tris-HCl pH 8 and 50 mM NaCl. The mixture was heated to 95°C for 30 s and allowed to cool to room temperature.

### RNA, RNA ligation reaction, and purification of full-length products

*Escherichia coli* tRNA^Lys^ and tRNA^fMet^ were purchased from Sigma-Aldrich. The RNA hairpin control was prepared by the Stanford PAN facility using the sequence 5′-phos-CGCGGGGUUUUUCCCCGCAACCA-3′. RNA substrates were ligated to adapters using RNA Ligase 2 (NEB). Ligation reactions were carried out in 20 μL of buffer recommended by the manufacturer supplemented with 0.5 mM ATP, 5% PEG 8000, and 2 μM each of RNA and adapter. To end the reaction and prepare the sample for purification, 50 μL urea loading buffer (7M urea and 0.1× TBE) was added to the sample and heated to 95°C for 5 min. The products were separated on a 7M urea PAGE gel in 1× TBE. The gel was post-stained with 2× SybrGold (Life Technologies) and product of appropriate size for the complete ligation product was excised. Ligated RNA was recovered by electroelution into 3.5 kDa MWCO D-tubes (Novagen) at 100 V for 2 h in 1× TBE. Recovered ligated RNA preparations were ethanol precipitated, quantified by Nanodrop (Thermo Scientific), and stored at −80°C.

### Nanopore experiments

Nanopore experiments were performed using a single α-HL nanopore (indicated by a step-wise increase in ionic current, range 68.0–72.5 pA) embedded in a planar 1,2-diphytanoyl-syn-glycero-phosphatidylcholine bilayer established on a ~25 μM aperture, as described previously (Akeson et al., 1999). Experiments were conducted in 0.3M KCl and 10 mM HEPES pH 8.0 at 28°C (± 0.4°C) at 180 mV (*trans* well positive). Dithiothreitol (2 mM final) and EDTA pH 8.0 (1 mM final) were added to the well on the *cis* side of the bilayer. In the case of experiments looking at the effect of Mg^2+^, MgCl_2_ was added to the buffer to 5 mM and EDTA was omitted from the *cis* side well. Nucleic acid substrates were added to 0.5 nM, unless otherwise noted to the *cis* well, and allowed to incubate 2 min to associate with the bilayer. For experiments where ϕ29 DNAP was to be added, an additional 12.5 min incubation was allowed; during this period, we observed captures of unbound RNA substrate prior to adding ϕ29 DNAP (Enzymatics) to 75 nM. Ionic current measurements were collected with an Axon Axopatch 200B (Molecular Devices) patch-clamp amplifier in whole-cell, voltage-clamped mode, and filtered with an analog low-pass Bessel filter at 5 kHz, then digitized using an Axon Digidata 1440A analog-to-digital converter (Molecular Devices) at 100 kHz bandwidth.

### Event detection and ionic current region measurements

For nanopore experiments that examined populations of ionic current blockade events, a custom developed event detection program was used (https://github.com/jmschrei/PyPore). The detection algorithm identified ionic current blockades that were self-terminating by selecting for segments that deflected from open nanopore current (68.0–72.5 pA) below a cutoff of 45 or 55 pA and with a minimum duration of >0.1 ms, where voltage was not reversed to eject the stand from the pore (current never <0 pA). For experiments where individual ionic current blockade events were examined, raw nanopore ionic current data were filtered with a digital 2 kHz low-pass Bessel filter and analyzed using Clampfit 10.4 (Molecular Devices). For ϕ29 DNAP molecular braking experiments, events were selected from current blockades that had durations >1 s and self-terminated. Event classification from adapted RNA(hairpin) substrates were analyzed as articulated in the text. For adapted tRNA data, complete translocation events were selected based on the criteria that they contained exactly two abasic-dependent regions; these were defined as high current regions with a mean current >33.5 pA, <36.5 pA, and minimum a duration >2 ms. After selection as complete translocation events, duration and mean current for states I–III were measured by hand using Clampfit’s internal statistics measuring program.

### Semi-logarithmic decision boundary and accuracy derivation

Event durations were transformed to log-durations (log_10_) and a linear decision boundary was established using the kernlab (v 0.9–19) package (Karatzoglou et al., 2004) under R (v 3.0.2). The ksvm parameters used were “type = ‘C-svc,’ kernel = ‘vanilladot,’ *C* = 10” to produce a soft-margin decision boundary. To assess classification accuracy, a fivefold training/test regimen was used. The data set was shuffled and then partitioned into five groups of nearly equal size. In a series of five tests, one of the five groups was withheld as a test set, while the decision boundary was calculated using the remaining four. This was repeated for each of the five groups. This procedure was repeated 50 times, providing 250 assessments of accuracy. Mean and standard deviation of these 250 accuracy scores are reported. For this study, we used a balanced accuracy score, calculated as the mean recall rate for each of the two data classes. For two classes with labels {1, −1},

$$\text{balanced accuracy}=\frac{\frac{\text{pred}\left(1\right)}{\text{true}\left(1\right)}+\frac{\text{pred}\left(-1\right)}{\text{true}\left(-1\right)}}{2}$$

where true(*n*) are counts of test data labeled *n*, and pred(*n*) are counts of test data that are correctly classified. The graphic provided in Figure 6 was derived using the full dataset and the kernlab package with parameters as above. Margins (dotted lines) in Figure 6 provide the optimized bounds that maximize the proper classification of labeled data outside the margin while minimizing the cost of misclassified data on the “wrong” side of that margin (Cortes and Vapnik, 1995).

## Results

### Capture and threading of tRNA through the α-HL nanopore is facilitated by ligation of an oligonucleotide adapter to the tRNA

Reading the nucleotide composition of individual tRNA molecules will require capture, denaturation, and threading of each strand sequentially through the nanopore. In our initial experiments, we found that native tRNA molecules caused long (>30 s) ionic current blockades of the α-HL pore (data not shown). These molecules had to be ejected by voltage reversal to re-establish an open-pore ionic current. This suggested that tRNA molecules in their native form would not readily translocate through the α-HL nanopore.

We reasoned that an extended single-stranded region, longer than the ACCA in native tRNA, may be needed to initiate threading of each tRNA molecule into the lumen of α-HL. To accomplish this, we devised a strategy to covalently attach synthetic nucleic acid strands to the 3′ and 5′ ends of the tRNA. This was achieved using a *Y-shaped*, partially double-stranded DNA–RNA adapter that contained a 3′ RNA overhang complementary to the universally conserved CCA tail in tRNA (Figure 1A). The strand of the adapter, which bares the 3′ RNA overhang, was designed to be ligated to the 5′ end of a tRNA (referred to as the “leading strand”). The unpaired region of the leading strand contained 35 single-stranded nucleotides and was designed to facilitate capture and threading into the nanopore. The double-stranded region of the adapter was designed to allow a dsRNA ligation, and effectively extended the tRNA adapter stem by 15 base-pairs.

The strand of the adapter that was designed to be covalently attached to the 3′ end of the tRNA (referred to as the “trailing strand”) incorporated a cholesterol tag at its 3′ end. This was designed to locally concentrate the adapted tRNA at the lipid bilayer-aqueous interface of the nanopore experimental setup. Association of the cholesterol moiety on the 3′ trailing strand with the bilayer should favor capture of the free 5′ end of the leading strand in the electric field surrounding the nanopore. Finally, the adapter design incorporated abasic residues into both leading and trailing strands to act as ionic current signal markers upstream and downstream of the ligated tRNA.

We initially tested if the adapter could be enzymatically ligated to tRNA using T4 RNA Ligase 2 (RNL2). Analysis of that ligation reaction revealed that a product of appropriate size (~160 nt) was generated only in the presence of both the adapter and a model tRNA substrate (*S. cerevisiae* tRNA^Phe^) (Figure 1B). These results indicated that enzymatic ligation with RNL2 was an effective method for adding adapter strands to this model tRNA. To test this further, we ran additional ligation experiments with *E. coli* tRNA^fMet^. This tRNA represented a more challenging substrate because it contains a non-canonical nucleotide pair at the end of the acceptor stem (RajBhandary, 1994). Our results showed that *E. coli* tRNA^fMet^ was also a reactive substrate for ligation to the adapter (data not shown).

**Figure 2:**
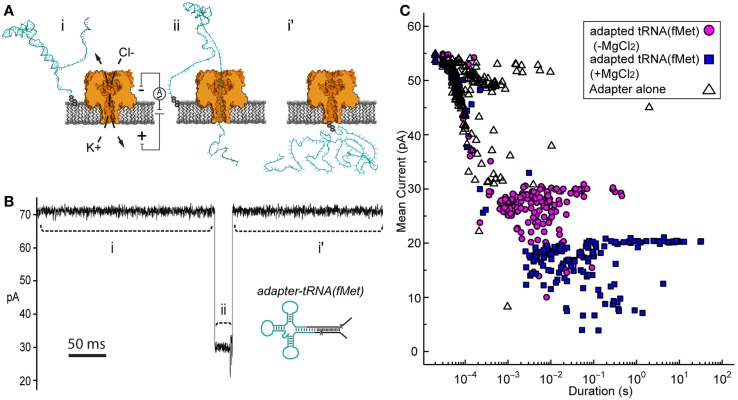
**Adapted tRNA-dependent ionic current blockades observed during single-channel α-HL nanopore experiments**. **(A)** Cartoon illustration of the single-channel nanopore apparatus and a proposed adapted tRNA translocation event. (i) A constant voltage (*trans* side+) is applied across a single α-HL nanopore (orange) embedded in a lipid bilayer (gray). (ii) Electrophoretic capture of an adapted tRNA (cyan) results in a decrease in the measured ionic current through the nanopore. (i’) Return to open channel current when the tRNA clears the pore in the *trans* compartment. **(B)** An ionic current trace from a nanopore experiment with adapted *E. coli* tRNA^fMet^ (inset). Ionic current regions i–ii and i’ in the trace (dashed lines) correspond to the proposed tRNA translocation event in **(A)**. The blockade event shown is typical of thousands of events observed during nanopore experiments with adapted tRNA^fMet^. Scale bar indicates 50 ms. **(C)** Nanopore blockade mean ionic current versus duration caused by adapted tRNA^fMet^ in the presence or absence of Mg^2+^. The mean current and duration of approximately 200 events are shown for representative nanopore experiments with either adapted tRNA^fMet^ (−Mg^2+^) (magenta circles) or adapted tRNA^fMet^ (+Mg^2+^) (blue squares). The adapter on its own (−Mg^2+^) (open triangles) was also examined as a negative control. In all cases, single-channel α-HL nanopore experiments were conducted at 180 mV (*trans* side+) with tRNA substrate at 0.5 nM in 0.3M KCl, 10 mM HEPES (pH 8.0), and ±5 mM MgCl_2_ (see Materials and Methods).

Initial nanopore experiments were performed with adapted *E. coli* tRNA^fMet^ using an established single-channel apparatus (see Materials and Methods) (Figure 2A). Ionic current blockade events were observed with a typical duration of tens of milliseconds (mean duration = 10^−2.6 ± 0.06^ s, variation of the mean shown as SEM) (Figures 2B,C, magenta circles). These events were longer than the events observed for the adapter alone, which were typically a millisecond or less (mean duration = 10^−3.9 ± 0.04^ s) (Figure 2C, open triangles). The increased duration of events with adapted tRNA^fMet^ suggested that longer or more structured molecules were being captured and translocated through the pore. The extremely short duration blockade events observed with the adapter alone were consistent with single-stranded nucleic acids being translocated through the pore (Deamer and Branton, 2002).

If in fact the longer dwell time was caused by tRNA, then conditions known to stabilize tRNA should increase event duration further. Magnesium ions are known to stabilize the tertiary fold of tRNA (Stein and Crothers, 1976; Serebrov et al., 2001). When we added magnesium chloride to the experimental buffer (5 mM final concentration), we observed an increase in event duration (100-fold or 2 Log_10_ units) relative to experiments absent Mg^2+^ (mean duration = 10^−1.4 ± 0.1^ s) (Figure 2C, blue squares). These blockades were self-terminating, as seen in experiments without Mg^2+^. This result was consistent with capture of a Mg^2+^-stabilized tRNA. Together, these results suggested that the electric-field driven denaturation of secondary, and potentially tertiary structure facilitated the translocation of tRNA through the pore.

### ϕ29 DNAP acts as a molecular brake during translocation of adapted RNA under non-catalytic conditions

In previous studies with both RNA and DNA, uncontrolled polynucleotide translocation rate through a nanopore was too high to resolve single nucleotide-level information about the translocating strand (Deamer and Branton, 2002). Therefore, we sought to slow the translocation of adapted tRNA to improve resolution of tRNA features and to provide definitive evidence that adapted tRNA molecules transit the pore in their entirety. Control of DNA transit rates using DNA polymerases has been documented, and Lieberman et al. (2010) showed that ϕ29 DNAP can serve as a “molecular brake” that controls the rate of DNA translocation through the α-HL pore under non-catalytic conditions (absent Mg^2+^ and dNTPs) (Lieberman et al., 2010). Furthermore, this molecular brake activity of ϕ29 DNAP has been observed on a chimeric DNA–RNA substrate with a nanopore device (J. Clarke, Oxford Nanopore Technologies, personal communication).

**Figure 3:**
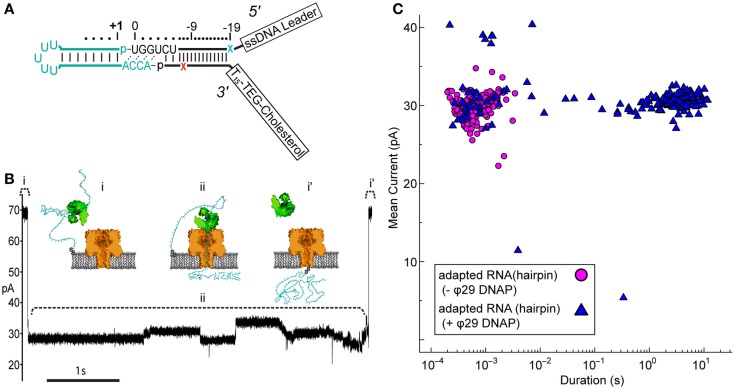
**Nanopore capture of adapted RNA complexed with non-catalytic ϕ29 DNAP**. **(A)** Schematic of the synthetic RNA(hairpin) construct (cyan) covalently attached to the adapter (black). This simple RNA hairpin is composed of a synthetic copy of the *E. coli* tRNA^fMet^ acceptor stem linked by a five uracil loop. This RNA was enzymatically ligated to the adapter as described previously (see Materials and Methods). Colored X’s indicate 5′ (blue) and 3′ (red) abasic residues in the adapter strands. **(B)** Representative ionic current trace during capture of an adapted RNA hairpin complexed with ϕ29 DNAP. ϕ29 DNAP (75 nM) and adapted RNA (0.5 nM) were added to *cis* side compartment which contained nanopore buffer absent Mg^2+^ and amended with 1 mM EDTA (see Materials and Methods). The cartoons above the ionic current trace represent proposed steps during ϕ29 DNAP-controlled translocation: (i) open channel prior to capture of the adapted RNA: ϕ29 DNAP complex; (ii) nanopore capture and translocation of adapted RNA bound to ϕ29 DNAP; (i’) return to open channel current when the RNA clears the pore into the *trans* compartment. Scale bar indicates 1 s. **(C)** Mean ionic current versus duration for adapted RNA(hairpin)-dependent blockades absent (magenta circles) or present (blue triangles) ϕ29 DNAP. Approximately 200 events are shown for each condition from a representative single experiment.

We wanted to determine if the ϕ29 DNAP molecular brake could also be used to control translocation of RNA molecules containing more complex structures, such as stem-loops found in tRNA. For this, we synthesized a simple RNA hairpin, which we ligated to the tRNA adapter (Figure 3A). This synthetic RNA [referred to as RNA(hairpin)] mimicked the acceptor stem of tRNA^fMet^, where the two halves of the acceptor stem were linked by a short loop region of five uracil residues.

Nanopore capture of complexes formed between the adapted RNA and ϕ29 DNAP, similar to those seen by Lieberman et al., should result in greatly increased dwell time of individual adapted RNA molecules within the aperture of α-HL. This would be observed as population of longer duration nanopore current blockades, which would be distinct from the shorter duration events of unbound adapted RNA strands.

Control nanopore experiments with the adapted RNA(hairpin) construct absent ϕ29 DNAP resulted in current blockade events with mean duration on the order of milliseconds (mean duration = 10^−3.2 ± 0.02^ s) (Figure 3C, magenta circles). Addition of ϕ29 DNAP to the buffer solution containing adapted RNA(hairpin) on the *cis* side of the nanopore apparatus produced two different types of current blockade events. These events typically fell into one of two populations: a short duration population (duration <0.1 s, mean 10^−2.9 ± 0.09^ s), similar to events in the control experiment, and a long duration population not seen in the control experiment (duration ≥0.1 s, mean 10^0.43 ± 0.03^ s) (Figures 3B,C, blue triangles). The shorter duration population (<0.1 s) appeared consistent with RNA(hairpin) absent ϕ29 DNAP and was statistically indistinguishable from the event population seen in the control (*p*-value <0.66, two-tailed *t*-test). The longer duration population (≥0.1 s) was longer in mean duration and was statistically different from the population seen in the control (*p*-value <0.0001, two-tailed *t*-test). This suggested that these long duration events were the result of ϕ29 DNAP binding the adapted RNA substrate and slowing strand translocation through the nanopore.

**Figure 4:**
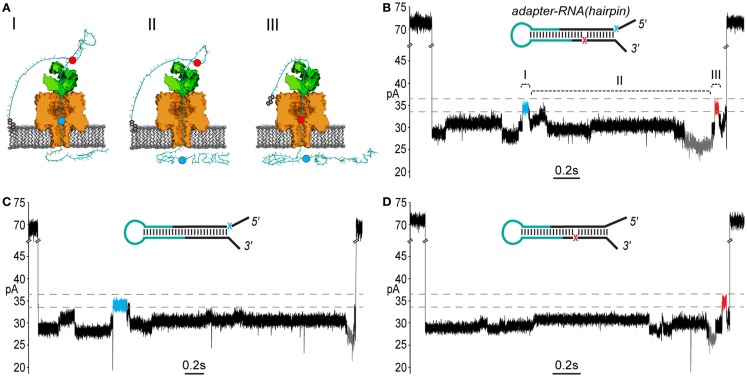
**End-to-end translocation of adapted RNA(hairpin) constructs through the α-HL nanopore**. **(A)** A cartoon model showing capture and translocation of an adapted RNA(hairpin) complexed with ϕ29 DNAP. The adapted RNA(hairpin) substrate is described in Figure 3. (I) Following capture of the 5′ end of the adapter strand, the polynucleotide translocates through α-HL until the leading 5′ abasic residue (blue circle) reaches pore limiting aperture. (II) Translocation continues through the RNA portion of the adapted molecule. (III) The trailing 3′ abasic residue (red circle) reaches the pore limiting aperture after translocation of the RNA(hairpin). **(B)** A nanopore ionic current trace during translocation of an adapted RNA(hairpin) (inset), which corresponding to the cartoon in **(A)**. The event displays two high current marker regions characteristic of the 5′ abasic residue (blue segment) and 3′ abasic residue (red segment) built into the adapter. Translocation events that contained both leading and trailing markers were observed in ~27% of events with durations exceeding 1 s. The marker ionic current level was 33.5–36.5 pA (dashed gray lines) under the experimental conditions used (see Materials and Methods). Scale bar indicates 0.2 s. **(C)** A nanopore ionic current trace during translocation of an adapted RNA(hairpin) (inset) bearing only the 5′ abasic residue (5′ mono-abasic adapted RNA). **(D)** A nanopore ionic current trace during translocation of an adapted RNA(hairpin) (inset) bearing only the 3′ abasic residue (3′ mono-abasic adapted RNA).

We included two abasic residues (1′-H deoxyribose) in the adapter strands near the ligation junctions with the RNA to act as indicators of strand translocation (see Figure 3A). Abasic residues have been shown to cause distinctive high current states that are apparent during enzyme-controlled translocation of oligonucleotides through the α-HL pore (Gyarfas et al., 2009; Lieberman et al., 2010). Because the abasic residues in the adapter (subsequently referred to as a “dual-abasic adapter”) flank the RNA insert, they should translocate through the nanopore before and after the RNA insert (Figure 4A). This should produce an ionic current trace with high current states bracketing an intervening region and indicate strand translocation occurred in a linear conformation. Further, the intervening region would correspond to the adapted RNA(hairpin) insert traversing the pore. As seen with DNA during ϕ29 DNAP-mediated translocation, the individual ribonucleobases of the extended chain should directly modulate the ionic current during translocation through the α-HL pore, resulting in a reproducible pattern of ionic current states (Cherf et al., 2012).

As predicted, translocation of dual-abasic adapted RNA(hairpin) constructs bound to ϕ29 DNAP resulted in blockade events containing two distinct ionic current states in the range of 33.5–36.5 pA that flank a reproducible pattern of ionic current (Figure 4B; Figure S1 in Supplementary Material). This suggested that the adapted RNA(hairpin) translocated completely through the nanopore, and that ϕ29 DNAP acted as a passive molecular brake for both the DNA and RNA portions of the chimeric molecule.

### The ionic current pattern produced by 5′ and 3′ abasic residues establishes the direction of translocation and indicates complete strand translocation

To establish the direction that ϕ29 DNAP-bound strands translocated through the nanopore, it was necessary to assign each of the observed high current states to either the 5′ or 3′ abasic residue. To do this, we synthesized adapters that contained only one of the 5′ or 3′ abasic residues. These “mono-abasic adapters” were ligated to the RNA(hairpin) substrate (see Figure 3A, cyan).

Substrates bearing the 5′ mono-abasic adapter typically produced events containing a single high current state (33.5–36.5 pA) near the beginning of the event (Figure 4C). Substrates bearing the 3′ mono-abasic adapter caused a similar high current state near the end of the event, which was preceded by a low current state (< = 26.5pA) (Figure 4D).

These observations provided an ionic current model for complete translocation of the dual abasic-adapted RNA(hairpin) in the 5′-to-3′ direction. That is, the 5′ abasic residue caused the first high current state, which was followed by the intervening current region corresponding to the RNA, which includes the low current state. This is followed by the high current state proximal to the end of the event that is caused by the 3′ abasic residue.

Using this model, we quantified the frequency of the leading and trailing high current states (henceforth referred to as leading and trailing markers) in events >1 s duration. Translocation of the dual-abasic adapted RNA resulted in 27.4% of events (102 of 372) that contained both the leading marker and trailing marker separated by a region containing a low current segment (Table 1). An additional 43% of events were classified as containing only a leading marker and 6.7% were classified as containing only a trailing marker. The disparity in frequency between leading and trailing markers suggested that the 3′ end of the strand was more difficult to resolve.

**Table 1:** **RNA(hairpin) nanopore translocation events classified by detection of leading and trailing high current markers**.

Substrate	Events	Leading and trailing high current observed^a^	Leading high current observed^b^	Trailing high current observed^c^	Other events^d^
RNA(hairpin) (dual-abasic adapter)	372	102 (27.4%)	160 (43.0%)	25 (6.7%)	85 (22.9%)
RNA(hairpin) (5′mono-abasic adapter)	287	3 (1.0%)	185 (64.5%)	10 (3.5%)	89 (31.0%)
RNA(hairpin) (3′mono-abasic adapter)	285	3 (1.1%)	4 (1.4%)	108 (37.9%)	170 (59.6%)

*^a^These events contained three required features as illustrated in Figure 4: (1) a high current marker segment (mean current 33.5–36.5 pA with ≥2 ms duration); (2) a low current segment (mean current = <26.5 pA with ≥10 ms); (3) a second high current segment proximal to the termination of the event*.

*^b^These events contained: (1) a single high current marker segment (mean current 33.5–36.5 pA with duration ≥2 ms); (2) a low current segment (mean current = <26.5 pA with ≥10 ms) that always followed the high current marker*.

*^c^These events contained: (1) a single high current marker segment (mean current 33.5–36.5 pA with duration ≥2 ms); (2) a low current segment = <26.5 pA with a duration of 10 ms) that always preceded the high current marker segment*.

*^d^Events classified as “other” included all events that could not be assigned to one of the other categories, such as those displaying more than two high current marker states and events displaying no high current marker segments*.

*All events were ≥1 s duration*.

The less frequent observation of the trailing marker relative to the leading marker suggested two possible explanations: either the 3′ abasic was absent from many strands, or that the translocation rate increased in the region of the 3′ abasic. In experiments with dual-abasic molecules, where both 5′ and 3′ abasic markers were resolved, the duration of the 3′ marker is notably shorter (Figure 4B; Figure S1A in Supplementary Material), supporting the latter hypothesis. Furthermore, in experiments with constructs containing mono-abasic adapters, we observed the same trends: strands bearing the 5′ mono-abasic adapter produced events with a single high current region 64.5% of the time with notably longer duration (Table 1 and Figure 4C; Figure S1B in Supplementary Material), and strands bearing the 3′ mono-abasic adapter produced a smaller fraction of events (37.9%) with a single high-current marker of shorter duration (Table 1 and Figure 4D; Figure S1C in Supplementary Material). This suggests that the translocation rate of the molecule increases after ϕ29 DNAP unzips the double stranded region. This would produce shorter duration ionic current signatures at the 3′ end. We reasoned that these shorter duration 3′ trailing markers were frequently unresolved at the measurement timescale used by our apparatus.

The result of the experiments using mono-abasic markers is consistent with 5′-to-3′ traversal of adapted RNA through the α-HL pore when bound to ϕ29 DNAP. By design, the 3′ end of adapted RNA would be retained in or at the lipid bilayer. This would preferentially leave the 5′ end of adapted RNA molecules available to initiate threading through the pore and act as the loading site for ϕ29 DNAP. By experiment, when a single abasic residue was present only on the leading strand of the adapter (5′), we observed a single long-duration high current marker toward the beginning of ϕ29 DNAP-bound translocation events. Conversely, inclusion of a single abasic residue only on the trailing strand of the adapter (3′) produced a single short-duration high current marker toward the end of ϕ29 DNAP-bound translocation events. Presence of both abasic residues in the adapter resulted in ~27% of ϕ29 DNAP-bound translocation events containing two high current markers. Taken together, these results support initiation of the ϕ29 DNAP-bound strand threading through the nanopore from the 5′ end, followed by the RNA portion, and terminating with the 3′ end of strand passing through and exiting the pore. A 5′-to-3′ voltage-driven “unzipping” process was previously observed for ϕ29 DNAP under non-enzymatic conditions on DNA (Lieberman et al., 2010). We conclude from the results presented here that: ϕ29 DNAP is being driven in the 5′–3′ direction, it unzips the dual-abasic adapted RNA(hairpin) strand, and in events where both markers were observed, the strand is translocated through the nanopore in its entirety. Further, these markers provide approximate boundaries of the RNA-dependent portion of the nanopore signal.

### *E. coli* tRNA^fMet^ and tRNA^Lys^ can be classified based on their nanopore current signals

If the ionic current segment flanked by the high current markers contains the RNA-dependent portion of nanopore signal, then that region should differentiate tRNA species. Further, a tRNA-specific change seen in the putative RNA-dependent region, when bordered by the adapter-dependent marker regions, would be evidence that the adapted tRNA translocate entirely through the nanopore. We used the ϕ29 DNAP-mediated braking method, as had been done with the RNA(hairpin) substrate, to improve temporal resolution of adapted tRNA. For this experiment, we selected two well-characterized tRNA species for nanopore analysis, *E. coli* tRNA^fMet^ and *E. coli* tRNA^Lys^. These tRNAs exist in the *E. coli* genome as either a single isoform (tRNA^Lys^) or as two isoforms that differ by only a single nucleotide (tRNA^fMet^) (RajBhandary, 1994). Additionally, tRNA^Lys^ and tRNA^fMet^ have similar lengths (76 and 77 nt, respectively), but have significantly different nucleotide compositions (50.0 and 64.9% G-C content, respectively), and would be expected to generate different ionic current signals.

**Figure 5:**
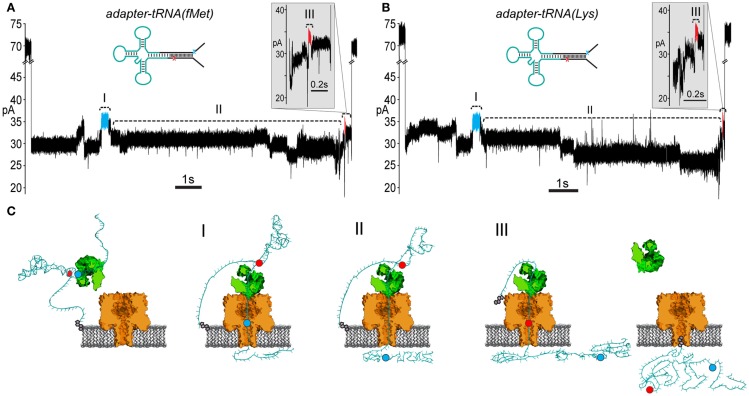
**Adapted *E. coli* tRNA^fMet^ and tRNA^Lys^ translocate through the α-HL nanopore**. **(A)** A representative nanopore ionic current trace for adapted *E. coli* tRNA^fMet^. The rate of translocation through the nanopore is controlled by ϕ29 DNAP (see Figure 3). The leading high current marker (I, blue segment) and the trailing high current marker (III, red segment) correspond to the 5′ abasic residue and 3′ abasic residue transiting the nanopore, as in Figure 4B. Region II of the trace contains the portion of the nanopore signal associated with tRNA translocation. Translocation events that contained both 5′ and 3′ current markers were observed in ~20% of all events with durations exceeding 1 s. Scale bar indicates 1 s. The inset shows a magnified view of the trailing 3′ high current marker. Scale bar indicates 0.1 s. **(B)** A representative trace from nanopore experiments with adapted tRNA^Lys^. Details are the same as in **(A)**. **(C)** A cartoon model of an adapted tRNA as it transits the α-HL nanopore. The panels to the left and right correspond to open channel before and after tRNA translocation (see Figure 3). Roman numerals correspond to the roman numerals in **(A,B)**. (I) Translocation of the leading adapter strand through α-HL until the 5′ abasic residue (blue circle) reaches the pore limiting aperture. (II) Translocation of the tRNA portion of the adapted molecule. (III) Translocation of the trailing adapter strand and 3′ abasic residue (red circle) through the pore limiting aperture after translocation of the tRNA.

Experiments with the adapted tRNA^fMet^ substrate produced 85 events that contained the leading and trailing markers bracketing an extended intervening current region (17.6% of 481 total events) (Figure 5A; Figure S2 in Supplementary Material). Experiments with the adapted tRNA^Lys^ substrate produced 77 events that contained the leading and trailing markers also bracketing an extended intervening current region (22.3% of 348 total events) (Figure 5B; Figure S2 in Supplementary Material). As suggested by the results with the adapted RNA(hairpin) substrate, these events were presumed to result from complete translocation of the adapted tRNA through the nanopore. These events were selected for further analysis of tRNA-specific ionic current signal.

**Figure 6:**
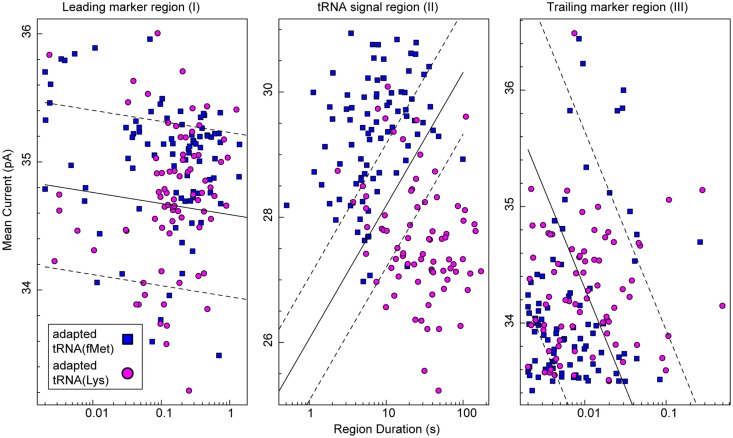
**Classification of tRNA molecules using duration and mean ionic for regions I–III of adapted tRNA translocation events**. We collected complete ionic current events (approximately 80 for each class) from experiments (*n* ≥ 5) using adapted *E. coli* tRNA^fMet^ (blue squares) or adapted tRNA^Lys^ (magenta circles). In our model (Figure 5C), regions I and III correspond to the common leading and trailing marker regions, respectively, and region II corresponds to the intervening RNA-dependent portion of the signal. The *x*-axis is the duration of a given region and the *y*-axis is mean current for that region. In each panel, the solid black line is a semi-logarithmic decision boundary established for that region using a soft-margin Support Vector Machine (SVM) (see Materials and Methods). SVM margins are shown as dashed lines. The associated classification accuracy for region II was 87.2 ± 5.3%. The associated classification accuracies for regions I and III were 60.0 ± 6.9 and 59.4 ± 7.0%, respectively. The SVM classification accuracy (mean and SD) was established using fivefold validation (see Materials and Methods). Data for the two tRNA species were collected separately from at least five independent nanopore experiments.

To test the putative RNA-dependent region for tRNA-specific signal (see Figures 5A,B, region II), we segmented the ionic current signal from each translocation event into regions I through III. As was the case for the RNA(hairpin) substrate (see Figure 4), regions I and III included the leading and trailing markers, respectively, which corresponded to the 5′ and 3′ abasic residues of the adapter translocating through the nanopore. These served as control regions for our analysis (Figure 5C). Bracketed by these markers, region II was expected to change based on the identity of the tRNA. Initial inspection of ionic current parameters, mean current, and dwell time suggested that Region II provided the best discrimination between tRNA^fMet^ and tRNA^Lys^ (Figure 6). As expected, mean current and duration for regions I and III did not appear to differ between tRNA^fMet^ and tRNA^Lys^.

To quantitatively assess the influence of tRNA type on ionic current, we analyzed the data in Figure 6 using a soft-margin support vector machine (SVM) (Cortes and Vapnik, 1995). A SVM was used to quantify the discrimination between the two tRNA species using their ionic current parameters in each of the three regions. The SVM produced an optimal linear decision boundary for the event log-durations and mean currents plotted in Figure 6. We used 5-way cross validation to calculate classification accuracy (mean and SD) of the boundaries produced for each of the regions (see Materials and Methods). Regions I and III provided discrimination between the two tRNA only slightly better than chance at 60.0 ± 6.9 and 59.4 ± 7.0% accuracy, respectively. In contrast, the putative tRNA dependent region II provided a classification accuracy of 87.2 ± 5.3%. This result demonstrated quantitatively that tRNA contributed to the nanopore signal in region II. Further, because the adapter-dependent regions I and III flanked region II, as they do in the adapted tRNA oligonucleotide strand, we concluded that adapted tRNA translocated completely through α-HL as an unfolded, linear strand in these instances.

## Discussion

In summary, we have shown that individual biological tRNA molecules can be unfolded and translocated through a nanopore as linear strands. To facilitate this, we developed a double-stranded oligonucleotide adapter that could be enzymatically ligated to biological tRNA. The two strands of the adapter acted to locally concentrate adapted tRNA at the bilayer and to initiate strand threading through the nanopore. Using ϕ29 DNAP under non-catalytic conditions, we were able to slow strand translocation through the pore. This allowed us to observe adapted tRNA translocating 5′-to-3′ through the pore as a linear strand when bound by ϕ29 DNAP. Finally, as evidence that tRNA influenced ionic current during translocation, we show that *E. coli* tRNA^fMet^ and tRNA^Lys^ produced differentiable ionic current signals.

Linear translocation of biological tRNA through the nanopore is a first step toward single-molecule direct sequence analysis of tRNA. Full implementation of a nanopore-based RNA sequencing method will require the following improvements: (1) *increased sensitivity of the nanopore*: for example, pores derived from mycobacterial species provide nucleotide-level sensitivity for DNA sequencing (Manrao et al., 2012). (2) *Regulation of tRNA movement in single nucleotide steps*: this may be accomplished by coupling an active molecular motor to translocate tRNA, as has been accomplished for DNA sequencing using ϕ29 DNAP in a catalytic mode (Laszlo et al., 2014). (3) *Correlation of ionic current states with nucleotide identity*: this has been achieved for canonical DNA bases and for various cytosine modifications (Manrao et al., 2012; Schreiber et al., 2013; Wescoe et al., 2014). (4) *Increased throughput*: for example, use of multi-channel nanopore devices will allow for reading tens of thousands of individual tRNA molecules.

Mitochondrial tRNA (mt.tRNA) genes comprise <10% of the mitochondrial genome (16,569 bp), yet 43% (245/571) of the currently known pathogenic mutations cataloged in MITOMAP are found in these genes (Brandon et al., 2005; Putz et al., 2007; Ruiz-Pesini et al., 2007). The lack of a direct view of transcribed mt.tRNA prevents use of sequence, modification state, and tRNA abundance as a facile diagnostic in mitochondrial pathology. We expect that a mature nanopore-based method for directly sequencing individual tRNA molecules will yield both canonical base identity and nucleotide modification states along entire strands. This will help advance our understanding of tRNA biology and the diagnosis of mitochondrial pathology as we fully appreciate the role of tRNA in human disease.

## Conflict of Interest Statement

The authors declare the following competing financial interest(s): Mark Akeson is a consultant to Oxford Nanopore Technologies. Patent application: SC2014_725_PRV, Molecular adapter for capture and manipulation of tRNA.

## Supplementary Material

The Supplementary Material for this article can be found online at http://journal.frontiersin.org/article/10.3389/fbioe.2015.00091
