# Supplementary material for: Capture, Unfolding, and Detection of Individual tRNA Molecules Using a Nanopore Device
Source: Front Bioeng Biotechnol. 2015 Jun 24;3:91. doi: 10.3389/fbioe.2015.00091 (PMC4478443; doi:10.3389/fbioe.2015.00091)
Supplement: Supplementary file 1 [file Data_Sheet_1.DOCX]

***Supplementary Material***

**Capture, unfolding, and detection of individual tRNA molecules using a nanopore device**

**Andrew M. Smith^1,2^, Robin Abu­Shumays^2^, Mark Akeson^2,3^, David L. Bernick^2*^**

## Supplementary Figures


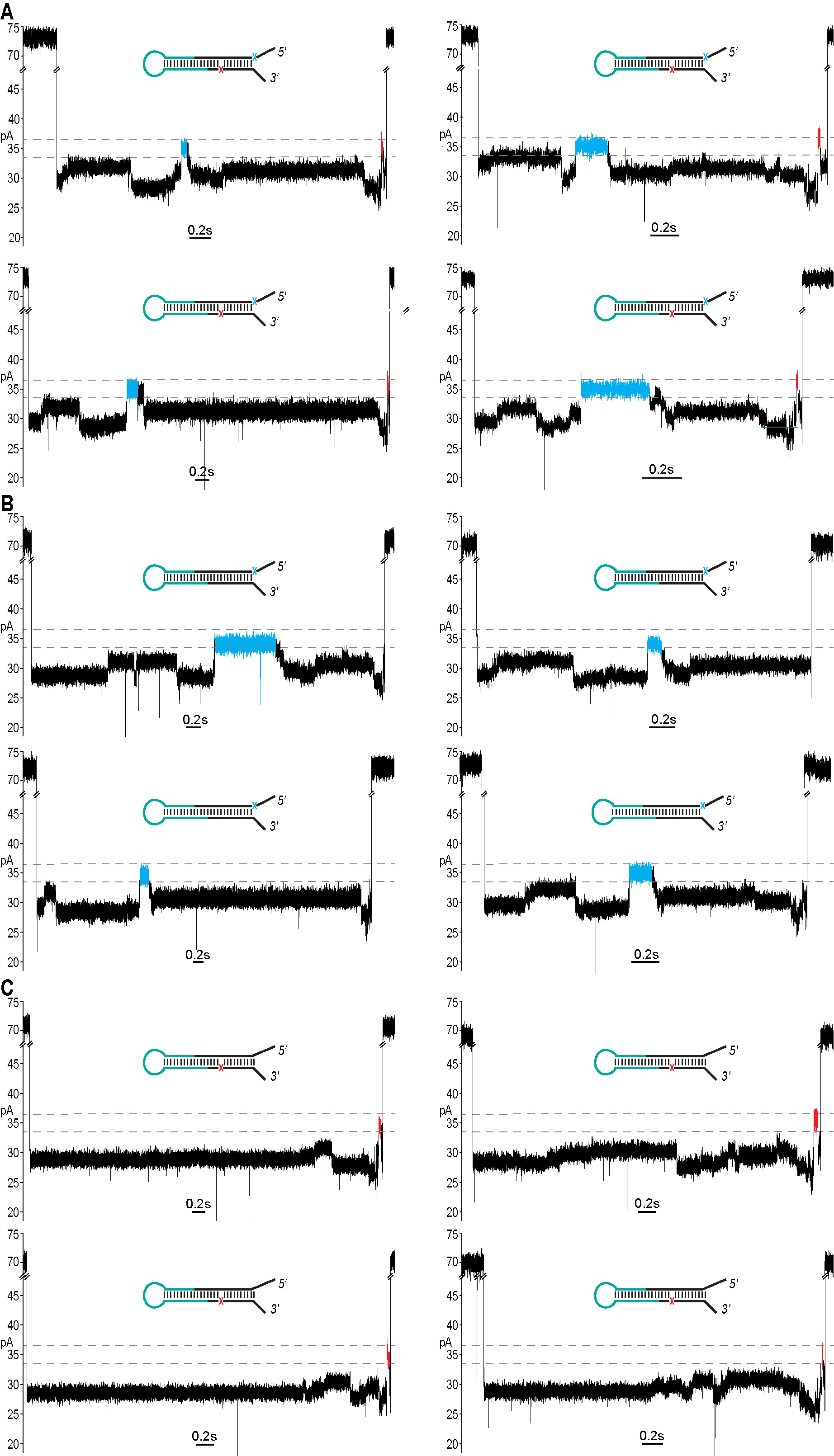


Supplementary Figure 1. Examples of ionic current traces during translocation of RNA hairpins translocation through α-HL with the dual abasic adapter, the 5′ mono-abasic adapter or the 3′ mono-abasic adapter. (A) Four examples of ionic current traces for the model RNA (hairpin) containing both the 5′ and 3 ′ abasic residues (as shown in Figure 4B). The rate of translocation through the nanopore is controlled by ϕ29 DNAP (see Figure 3). The events display two high current marker regions characteristic of the 5′ abasic residue (blue segment) and 3′ abasic residue (red segment) built into the adapter. Scale bar indicates 0.2 seconds. (B) Four examples of adapted RNA hairpin containing the 5' abasic residue displaying the leading high current marker (blue segment) (as shown in Figure 4C). Scale bar indicates 0.2 seconds. (C) Four examples of adapted RNA hairpin containing the 3' abasic residue displaying the trailing high current marker (red segment) (as shown in Figure 4D). Scale bar indicates 0.2 seconds.


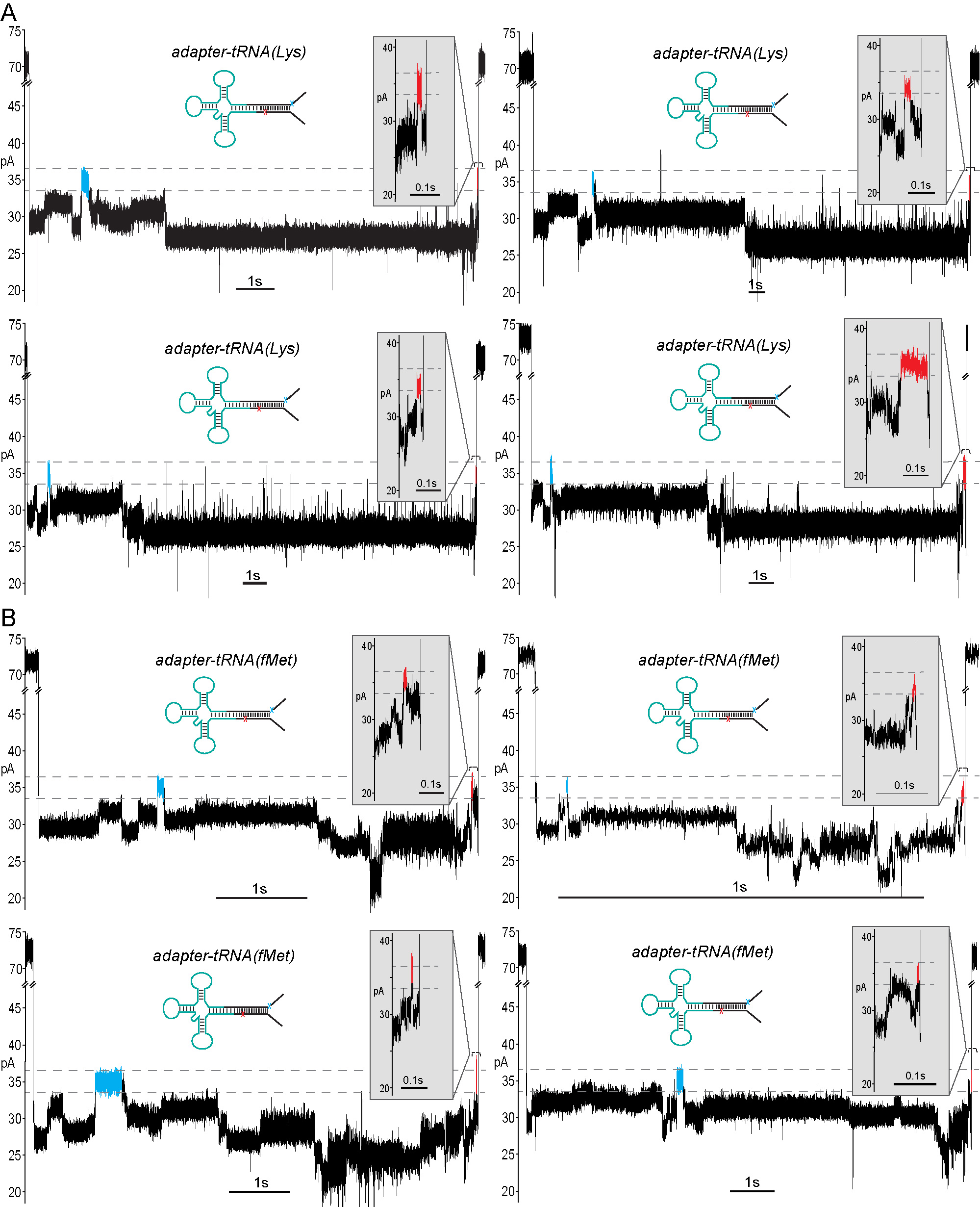


Supplementary Figure 2. Examples of adapted tRNA^Lys^ and tRNA^fMet^ ionic current traces during ϕ29 DNAP-regulated translocation through α-HL nanopore. (A) Four ionic current traces during translocation of adapted tRNA^Lys^ molecules are shown. The rate of translocation through the nanopore is controlled by ϕ29 DNAP. The leading high current marker is highlighted in blue and the trailing high current marker is highlighted in red (as in Figure 5B). Scale bar indicates one second. The inset shows a magnified view of the trailing high current marker. Scale bar indicates 0.1 second. (B) Four examples of adapter-tRNA (fMet) events are shown. The leading high current marker is highlighted in blue and the trailing high current marker is highlighted in red (as in Figure 5A). Scale bar indicates one second. The inset shows a magnified view of the trailing high current marker. Scale bar indicates 0.1 second.
